# Comparing outcomes of transperineal to transrectal prostate biopsies performed under local anaesthesia

**DOI:** 10.1002/bco2.112

**Published:** 2021-10-09

**Authors:** Kelven Weijing Chen, Gregory Pek, Qiao Yufei, Poh Choo Toh, Nicholas Kuek, Joe King Chien Lee, Lincoln Guan Lim Tan, Woon Chau Tsang, Edmund Chiong

**Affiliations:** ^1^ Department of Urology National University Hospital Singapore; ^2^ Department of Surgery National University of Singapore Singapore

**Keywords:** biopsy, local anaesthesia, PrecisionPoint, prostate, transperineal, transrectal

## Abstract

**Objectives:**

To compare and review the outcomes of transperineal (TP) prostate biopsies with transrectal (TR) biopsies performed under local anaesthesia (LA). A review of the relevant published literature is presented.

**Patients and methods:**

We prospectively analysed 212 consecutive patients who underwent TP prostate biopsy using the PrecisionPoint™ access system under LA, at our institution from October 2018 to March 2020. We compared the morbidity and cancer detection rates using this approach with our historical cohort of 178 patients who underwent the TR biopsy method under LA.

**Results:**

The mean age of the TP biopsy group was 69 years, and median prostate specific antigen (PSA) was 13.17 ng/ml. Mean prostate volume was 45.1 ml with a median of 12 cores taken per patient. Patient demographics were similar to our TR biopsy cohort, with mean age of 68 years, median PSA of 10.76, mean prostate volume of 49.6 ml and a median of 12 cores taken per patient. The TP biopsy group had 0% sepsis rate compared with 2.2% in the TR group. Haematuria in the TP versus transrectal ultrasonography (TRUS) cohort was 0.9% versus 1.7%, respectively. The TP biopsy‐naïve group had a cancer detection rate of 63.5% (127 of 200 patients), of which 84% were ≥Grade Group 2 (GG2). The TR biopsy‐naïve group had cancer detection rate of 50% (86 of 172 patients), of which 87.2% was ≥GG2.

**Conclusion:**

TP prostate biopsy had less urinary infectious and septic complications compared with the TR approach. Our data suggest at least comparable diagnostic accuracy between both biopsy approaches.

## 1 INTRODUCTION

Prostate cancer is the second most frequent cancer diagnosis made in men and the fifth leading cause of death worldwide.^1^ Apart from the utility of digital rectal examination (DRE), diagnostic imaging modalities and prostate specific antigen (PSA) as investigative tools, histology remains the gold standard in the diagnosis of prostate cancer. Prostate biopsies may be performed via the transperineal (TP) or the transrectal (TR) route. The recent systematic review and meta‐analysis by Xiang et al.^2^ comparing TP and TR prostate biopsies revealed comparable diagnostic accuracies between the two routes.

TR route of prostate biopsies remains the most common approach. One major drawback is the uncommon but serious risk of sepsis.^3^ Infective complications may be severe and contribute to increased hospital admissions. In an analysis by Jiang et al.,^4^ 38% of patients who presented with sepsis had received correct prophylactic antibiotics, highlighting the inherent difficulty in predicting or preventing this potentially disastrous complication. As the use of prophylactic antibiotics becomes routine, the emergence of fluoroquinolone‐resistant organisms has also increased.^5^ The TP route of prostate biopsy greatly reduces this risk of infection as the needle traverses the perineal skin rather than the rectal mucosa.^6^ Contemporary series have reported infective complications as low as 0–1%, even without the use of prophylactic antibiotics.^7^, ^8^

The peripheral zone (PZ) contains the majority of prostatic glandular tissue. The PZ is mostly located at the back of the gland, closest to the rectal wall, and extends to the apical portion of the gland. About 70–80% of prostate cancers originate in the PZ. Earlier studies^9^, ^10^ have shown improved cancer detection rate in the apical and anterior region of the prostate with the TP route. The Victorian Transperineal Biopsy Collaboration^11^ showed that up to 75% of cancers involved the anterior region on second biopsy, whereas 25% were confined exclusively within the anterior region. In this study, we compare the outcomes of our centre's experience with TP prostate biopsies under local anaesthesia (LA) and compare it with our prior series of TR biopsies. We also present a review of current literature on the outcomes of a contemporary series of TP biopsies under LA.

## 2 PATIENTS AND METHODS

The primary objective of this study was to evaluate the safety and efficacy of the TP biopsy under LA. Study ethics was obtained and approved through our institutional review board. We prospectively evaluated 212 consecutive patients who underwent TP biopsy under LA at our institution from October 2018 to March 2020. We compared the complications and cancer detection rates using this approach with our historical cohort of 178 patients who underwent traditional TR biopsy from July 2017 to January 2019. As upfront prostate magnetic resonance imaging (MRI) was not yet a standard recommendation for biopsy‐naïve men at the point of initiation of our study, our patients were given the option of either going for prostate biopsy versus having a prostate MRI first.

**FIGURE 1:**
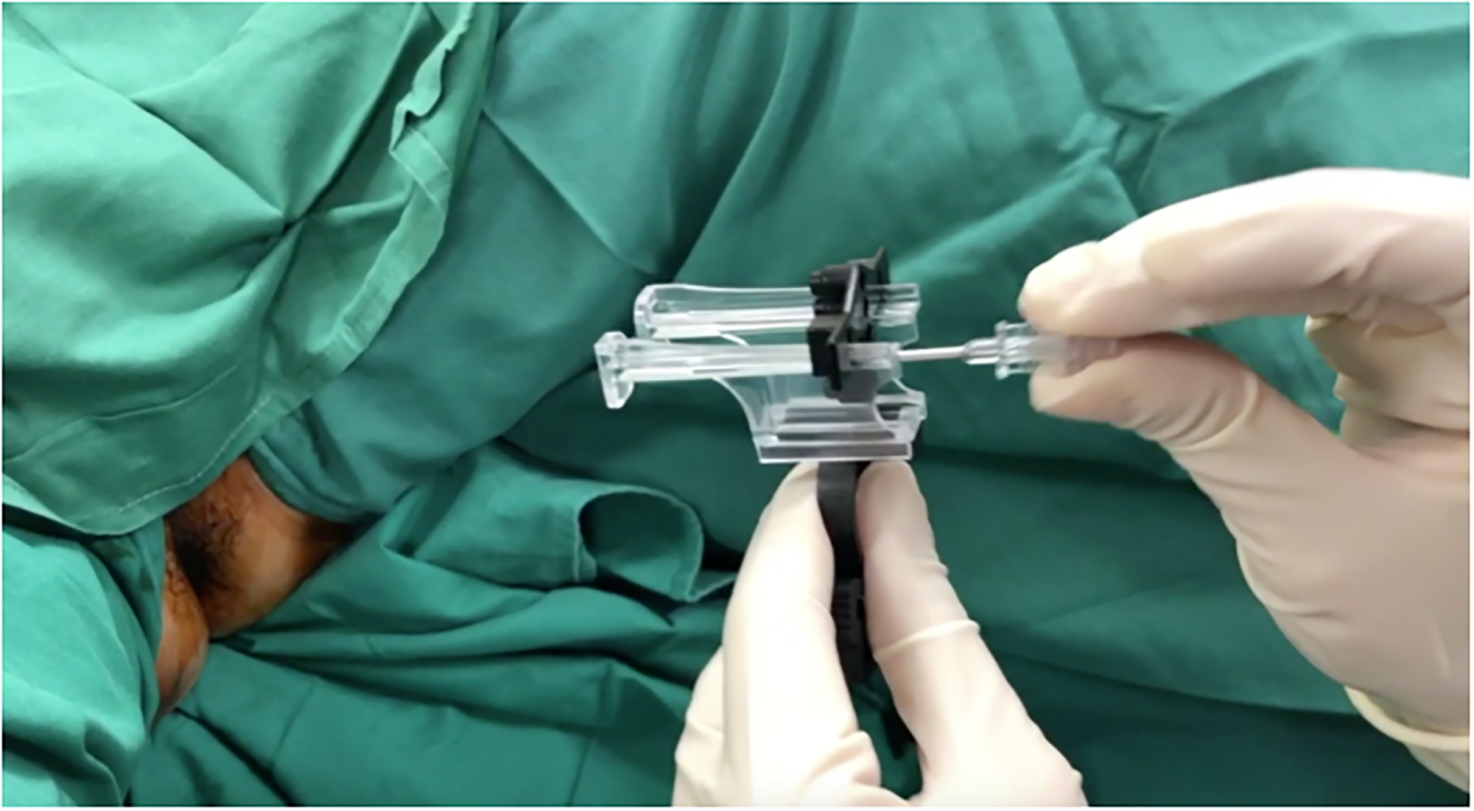
The PrecisionPoint™ Transperineal Access System

**FIGURE 2:**
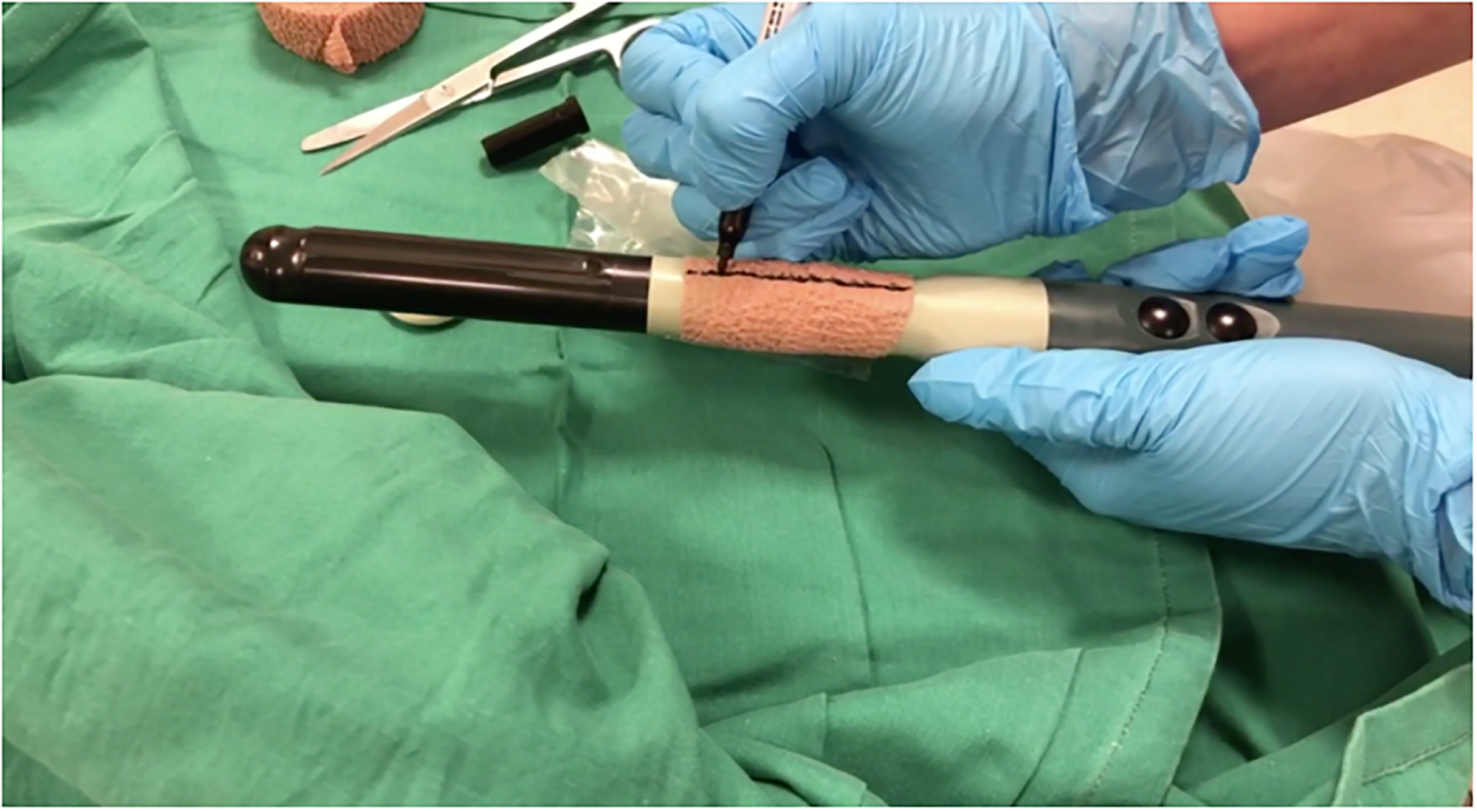
Wrapping transrectal probe with Coban™ bandage

The TP biopsies were performed with the PrecisionPoint™ Transperineal Access System with a BK Medical® Endocavity 8848 transrectal probe. Our patients were routinely given a dose of oral cephalexin 500 mg on the day of the TP biopsy. Twelve patients (5.6%) were not given prophylaxis, and this was based on surgeon preference. The PrecisionPoint™ Transperineal Access System has a rail/clamp subassembly, a needle carriage with 4 apertures and a 15‐gauge access needle (Figure 1). This is a single‐use item and is disposed after use. The assembled device is then clamped to the BK Medical® Endocavity 8848 transrectal probe. We wrapped the proximal end of the TR probe with adhesive Coban™ bandage before attaching the clamp of the PrecisionPoint™ TP access system (Figure 2). The bandage improves the grip of the clamp onto the probe, thereby minimising rotational movement of the PrecisionPoint™ TP access system during the biopsy. We marked the 12 o'clock position on the bandage with a marker to guide alignment with the ultrasound transducer.

**FIGURE 3:**
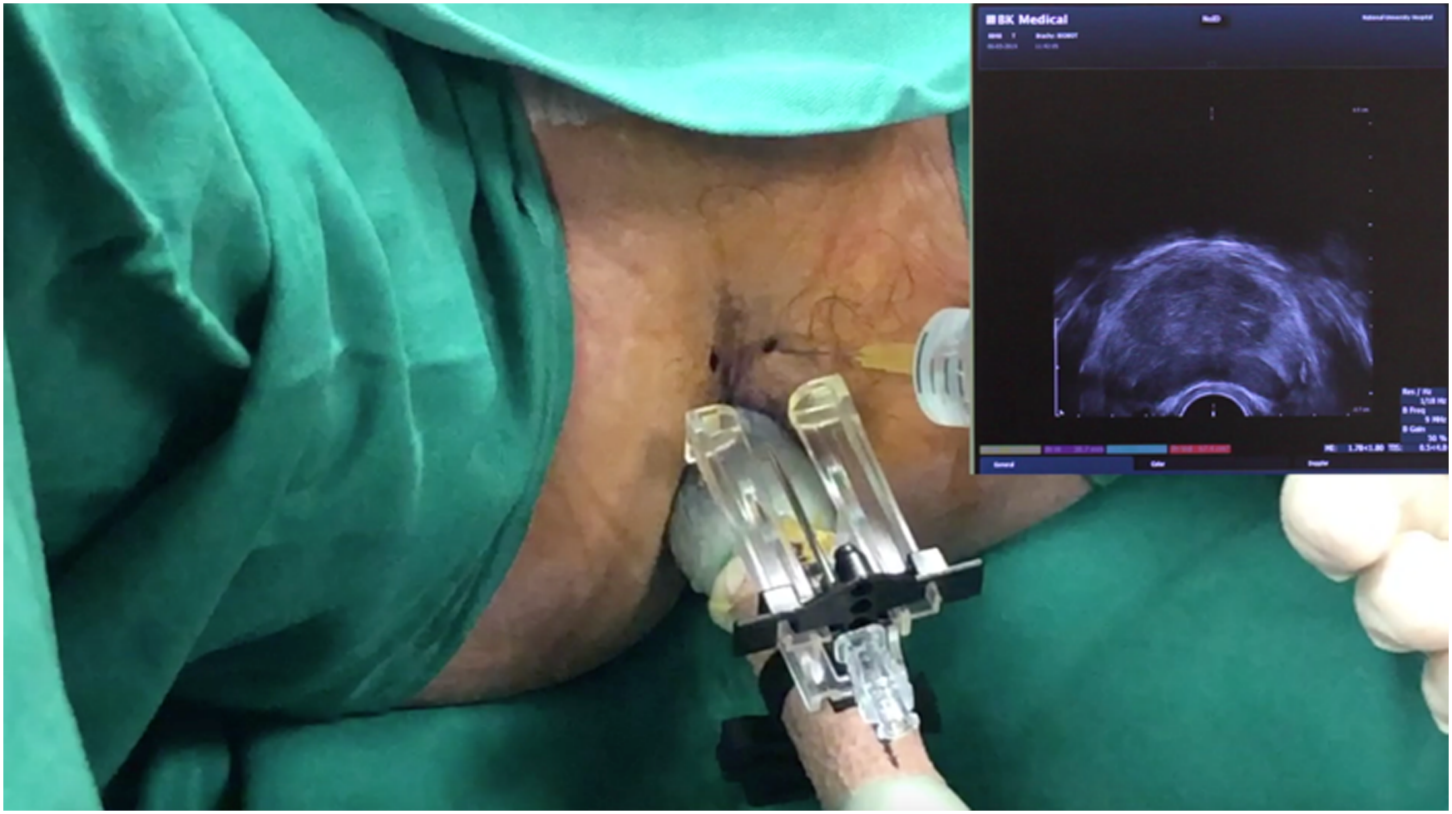
Injection of local anaesthesia (LA) at perineal skin

**FIGURE 4:**
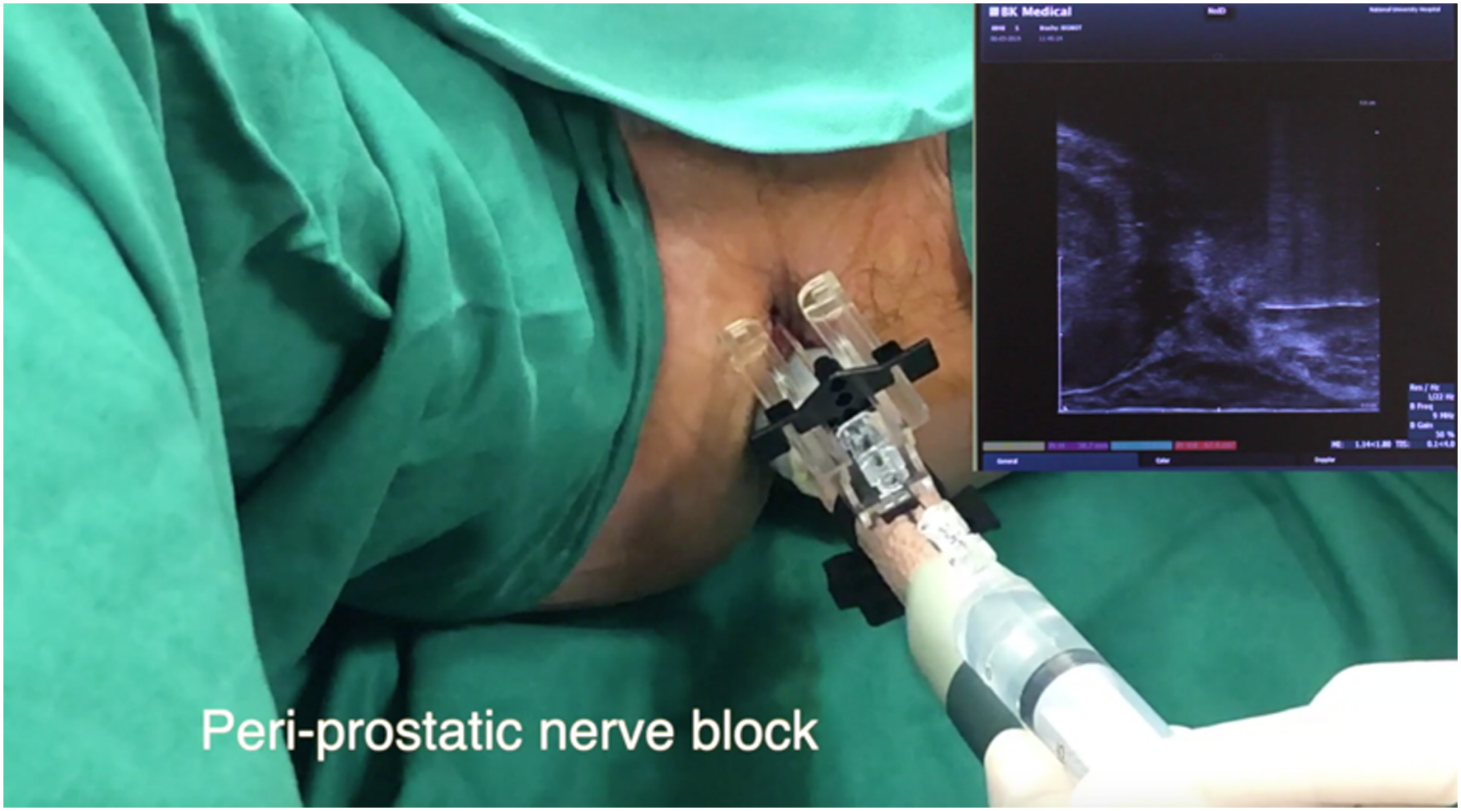
Injection of local anaesthesia (LA) as periprostatic nerve block

**FIGURE 5:**
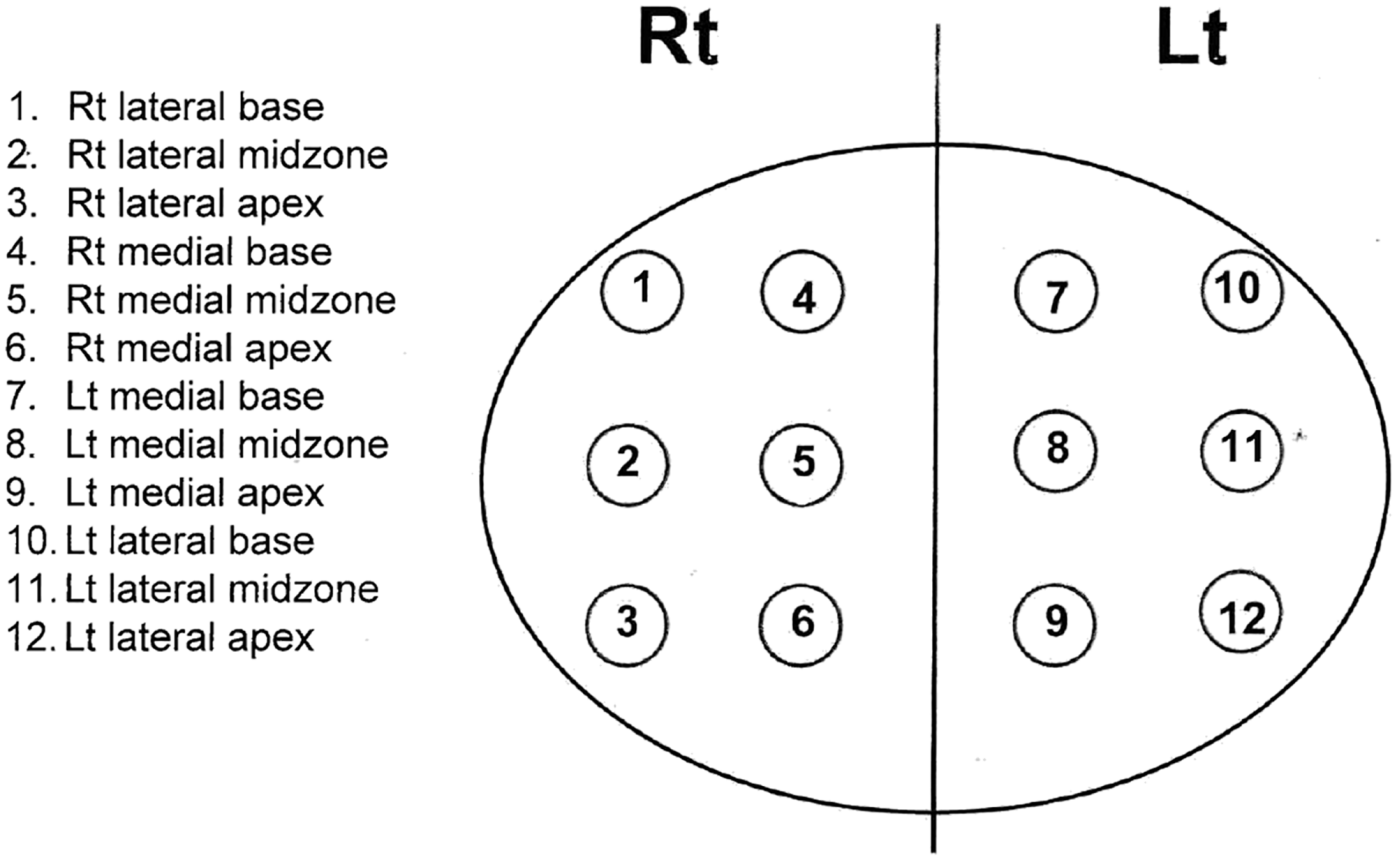
Systematic 12‐core biopsy template

The patients are placed in lithotomy position with heel stirrups. The scrotum is lifted away from the perineum with tape. The perineum is cleaned with povidone iodine and the ultrasound probe is inserted into the rectum to visualise and measure the prostate. We injected 1% lignocaine superficially at the perineal skin on both sides prior to inserting the access needle sheath. Further, 10‐ml 1% lignocaine was given on each side as periprostatic nerve block (Figures 3 and 4). Systematic 12 core biopsies were taken based on a template (Figure 5). Additional target cores were taken at the discretion of the physician. Saturation biopsy, if done, was performed based on the Ginsburg protocol.^11^ Procedural pain score was recorded using visual analogue scores (VAS) from patients immediately after the procedure.

In our TR biopsy cohort, we gave prophylactic oral fluroquinolones 1 day before and up to 3 days after the biopsy, with an additional single intramuscular gentamicin injection preprocedure. Dulcolax was given for bowel preparation the night before the biopsy. The rectum was cleansed with cotton buds soaked with chlorhexidine with aid of a proctoscope prior to biopsy. TR biopsy was postponed if persistent soiling of gauze was observed after cleaning.

Patient demographics, prostate size, PSA and DRE findings were collected prospectively. Number of cores of biopsy taken, cancer detection and complications rates were analysed. Urosepsis was defined as at least 2 out of 4 systemic inflammatory response syndrome (SIRS) criteria with a proven infection. In the subanalysis, patients were further stratified into three groups based on serum PSA levels (PSA < 10, PSA 10–20, PSA > 20). All statistical analyses were performed using GraphPad Prism (GraphPad Software, Inc., CA, USA) including student *t* test, chi‐square test and Mann–Whitney tests. A *p* value of <0.05 was considered significant.

## 3 RESULTS

### 3.1 Demographics

All 212 patients successfully underwent TP biopsy under LA. The mean age of patients who underwent TP biopsy was 69.40 ± 7.75, with a median PSA of 13.17 ng/ml (6.82–47.13). Mean prostate volume was 45.08 ml (29.0–54.4), and the mean and median number of cores taken per biopsy was 12.94 ± 3.069 and 12 (4–38), respectively. The wide range in biopsy cores was due to 13 patients (6.13%) having undergone saturation biopsy in the TP group. Both TP and TR groups were well matched (P═NS) when comparing preprocedure demographics including age, median PSA, PSA density, prostate volume and DRE findings (Table 1).

**TABLE 1:** Characteristics of transperineal and transrectal prostate biopsy

	Transperineal biopsy (TPBX) *n* = 212	Transrectal biopsy (TRUS) *n* = 178	*p* value
Age (years)	69.40 ± 7.75	68.24 ± 7.98	0.1469^a^
Median PSA (IQR 25–75%)	13.17 (6.82–47.13)	10.76 (6.45–50.97)	0.6199^c^
Median PSA density (IQR 25–75%)	0.29 (0.17–1.17)	0.27 (0.14–1.10)	0.2189^c^
Prostate volume (IQR 25–75%)	45.08 ± 26.78 (29–54.40)	49.62 ± 27.76 (32–62)	0.0891^a^
DRE (abnormal)	102/205	77/177	0.2188^b^
Median biopsy core Number (IQR)	12 (4–38)	12 (2–18)	<0.0001^c^
Mean biopsy core Number (IQR)	12.94 ± 3.069 (4–38)	11.14 ± 2.92 (2–18)	<0.0001^c^
VAS pain score (IQR)	3.67 ± 2.57 (0–9)	‐	

*Note*: Value expressed as mean ± SD unless stated.

Abbreviations: DRE, digital rectal examination; IQR, interquartile range; PSA, prostate specific antigen; VAS, visual analogue scale.

^a^ Student *t* test.

^b^ Chi‐square test.

^c^ Mann–Whitney test.

### 3.2 Complications

There was no reported case of urosepsis (Table 2) in the TP compared with the TR group (0% vs. 2.2%, *p* = 0.04). The rate of nonseptic urinary tract infection (UTI) complications in the TP biopsy group was also lower than the TR group (0.9% vs. 2.2%). Of the two patients in the TP group who reported UTI symptoms, one had a previous background of chronic bacterial prostatitis. One patient (0.9%) in the TP group had symptomatic hypotension, which was attributed to vasovagal in nature. He did not show signs of sepsis, had a normal urine microscopy and urine culture yielded no growth. He was hospitalised for monitoring and discharged after 1 day. Two patients in the TP group (0.9%) reported mild gross haematuria but did not require hospitalisation or intervention. The urinary retention rate was 3.8% for the TP group compared with 4.5% in the TR group (*p* = 0.8). The mean VAS score was 3.67 ± 2.57 (0–9) in the TP group.

**TABLE 2:** Prostate biopsy complications

	TP biopsy *n* = 212	TR biopsy *n* = 178	*p* value^a^
Acute urinary retention	8 (3.8%)	8 (4.5%)	0.8008
Haematuria	2 (0.9%)	3 (1.7%)	0.6640
Vasovagal	1 (0.9%)	0	1.0000
UTI	2 (0.9%)	4 (2.2%)	0.4189
Sepsis	0	4 (2.2%)	0.0431
Overall complication rate	13 (6.1%)	20 (11.2%)	0.0993

Abbreviations: TP biopsy, transperineal biopsy; TR biopsy, transrectal biopsy; UTI, urinary tract infection.

^a^ Chi‐square test.

### 3.3 Use of MRI prostate

Sixty‐three of 212 (29.7%) patients who underwent TP biopsy had an MRI prostate done before the biopsy (Table 3). Of these 63 patients who had an MRI prostate, 39 patients had PIRADS 4 or 5 lesions on MRI. They subsequently underwent cognitive TP biopsy, as they had declined to undergo MRI‐US fusion targeted plus systematic biopsy using another platform. The clinically significant (group grade [GG] ≥ 2) cancer detection rate was 50% and 93% for PIRADS 4 and 5 lesions, respectively. Fifteen patients did not have any lesions (PIRADS < 3) detected on MRI prostate, but two were found to have clinically significant cancer on TP biopsy, giving a false negative rate for MRI prostate of 13%. In the historical TR biopsy cohort, only a handful of patients had an MRI prostate done before biopsy.

**TABLE 3:** TPBX + MRI versus TPBX only

	TP biopsy + MRI prostate *n* = 63	TP biopsy only *n* = 149	*p* value^a^
No cancer	22 (34.9%)	59 (40.0%)	0.5241
Insignificant cancer	6 (9.5%)	15 (10.1%)	0.9042
CS cancer GG ≥ 2	35 (55.6%)	75 (50.3%)	0.4893

Insignificant Cancer: Group Grade 1.

Abbreviations: CS Cancer GG ≥ 2, clinically significant cancer group grade ≥2; MRI, magnetic resonance imaging; TPBX, transperineal biopsy.

^a^ Student *t* test.

### 3.4 Cancer detection rate

Two hundred of 212 patients (94.3%) in the TP group and 172 of 178 patients (96.6%) in the TR group were biopsy‐naïve. When comparing only the biopsy‐naïve patients, cancer detection was 63.5% (127 of 200) compared with 50% (86 of 172) in the TP and TR groups, respectively (*p* = 0.012). Clinically significant cancer (≥Gleason grade 2) was detected in 53.5% (107 of 200) of patients in the TP group, compared with 43.6% (75/172) in the TR group (*p* = 0.062), which suggested a trend towards higher detection rates in the TP group (Table 4).

**TABLE 4:** Cancer detection rate for biopsy‐naïve patients

	Transperineal biopsy (TPBX) *n* = 200	Transrectal biopsy (TRUS) *n* = 172	*p* value
Cancer detection rate	127/200 (63.5%)	86/172 (50%)	0.0115^a^
Cancer detection rate (GG ≥ 2)	107/200 (53.5%)	75/172 (43.6%)	0.0617^a^
Anterior core positive rate	99/200 (49.5%)		
Cancer in anterior cores (GG ≥ 2)	83/200 (41.5%)		

Abbreviations: Cancer detection rate GG ≥ 2: Cancer detection rate group grade ≥2; cancer in anterior cores GG ≥ 2, cancer in anterior cores group grade ≥2.

^a^ Chi‐square test.

Upon subanalysis of both biopsy‐naïve groups based on PSA ranges (Table 5), patients with PSA < 10 had a higher cancer detection rate when utilising the TP (45.1%) compared with TR (23.3%) route of biopsy (*p* = 0.0034). However, this did not translate into a higher detection of clinically significant cancers (GG ≥ 2, *p* = 0.17) for this subgroup of patients. There was no difference in cancer detection rates for TP and TR groups when stratified by PSA 10–20 (*p* = 0.797) or PSA > 20 (*p* = 1.00). In the TP biopsy‐naïve group, 49.5% of patients had anterior cores positive for cancer, of which 83.8% were clinically significant.

**TABLE 5:** PSA stratified clinically significant cancer detection rate for biopsy‐naïve patients

	PSA < 10		PSA 10–20		PSA > 20	
	TPBX	TRBX	TPBX	TRBX	TPBX	TRBX
Cancer detection rate	37 (45.1%)	20 (23.3%)	19 (50.0%)	11 (44.0%)	71 (88.8%)	55 (90.2%)
*p* value^a^	0.0034		0.7971		1.0000	
CS cancer GG ≥ 2	20 (24.4%)	13 (15.1%)	17 (44.7%)	8 (32.0%)	70 (87.5%)	54 (88.5%)
*p* value^a^	0.1737		0.4308		1.0000	
CS cancer GG ≥ 3	8 (9.8%)	7 (8.1%)	13 (34.2%)	7 (28.0%)	64 (80.0%)	52 (85.3%)
*p* value^a^	0.7913		0.7829		0.5070	

Abbreviations: CS cancer GG ≥ 2, clinically significant cancer group grade ≥2; CS cancer GG ≥ 3, clinically significant cancer group grade ≥3; PSA, prostate specific antigen; TPBX, transperineal biopsy; TRBX, transrectal biopsy.

^a^ Chi‐square test.

## 4 DISCUSSION

The TP route of prostate biopsy has been gaining popularity recently, largely due to its potential lower risk of septic complications. A study by Stefanova et al.^7^ that analysed 1287 patients who had undergone TP prostate biopsy had shown the feasibility of this approach as compared with the TR route of biopsy. The PrecisionPoint™ Transperineal Access System has been used to aid prostate biopsy.^9^, ^12^ Our institution's data serve to further validate the safety of this TP approach under LA and show that it is able to achieve at least comparable prostate cancer detection rates when compared with TR biopsies.

Previous population‐based studies have shown severe sepsis rates at 0.1–3.5% after TR biopsy. Management of such infectious complications causes significant financial burden on healthcare systems.^13^ Our TR biopsy cohort had a 2.2% rate of severe sepsis requiring hospitalisation and intravenous antibiotics. In comparison, none of the patients in our TP cohort developed sepsis. This was consistent with other reported experiences of TP prostate biopsy under LA. We also saw a lower rate of nonseptic UTI complications for TP compared with TR biopsy (0.9% vs. 2.2%). This is comparable with other centres' UTI rate, ranging from 0% to 2.2% in Huang's study (Table 6). Given the low risk of septic complications, some centres have even moved to omission of peri‐procedural prophylactic antibiotics. Ristau et al.^8^ reported 1000 patients in his series, giving a single‐dose cephalexin in the first 600 patients and no antibiotics in the next 400 patients with no culture proven UTIs and no hospital admissions for sepsis. Another report by Meyer et al.^9^ showed that no patients experienced an infectious complication despite omission of peri‐procedural antibiotics in all cases following a TP biopsy. The reduction in use of prophylactic antibiotics would help to limit the development of fluoroquinolone‐resistant organisms worldwide.

**TABLE 6:** Freehand LA‐TP complications

Freehand LA‐TP Bx	AUR	Haematuria	UTI	Urosepsis	Pain (VAS)
Hong Kong ‐ Lo et al.^14^	3.0%	0%	0%	0%	‐
London, UK ‐ Kum et al.^12^	0.6%	0.6%	0%	0%	3.6 (2.75 in clinic, 4.5 in day surgery unit)
Taiwan ‐ Huang et al.^15^	3.0%	5.3%	2.2%	0%	4 (3–5)
USA (Johns Hopkins) ‐ Meyer et al.^9^	4.7%	2.3%	0%	0%	‐
USA (University Connecticut) Ristau et al.^8^	0.1%	0%	0.3%	0%	‐
Toronto, Canada ‐ Stefanova et al.^7^	1.6%	0%	0.3%	0%	2.75 (LA injection 3.1, periprostatic injection 3.0, biopsy‐taking 2.5, probe insertion 2.4)
Cambridge, UK ‐ Thurtle et al.^16^	0%	66.7%	0%	0%	3.12 (initial DRE 2.04, probe insertion 2.97, LA injection 2.67, biopsy‐taking 1.83)
Singapore ‐ Chen et al.^17^	3.8%	0.9%	0.9%	0%	3.67 ± 2.57 (0–9)

Abbreviations: DRE, digital rectal examination; LA‐TP, local anaesthesia‐transperineal; UTI, urinary tract infection; VAS, visual analogue scale.

There was a higher overall detection rate (63.5% vs. 50%, *p* = 0.012) and a trend towards more clinically significant prostate cancers (53.5% vs. 43.6%, *p* = 0.062) being detected when comparing our TP versus TR biopsy‐naïve cohort. Our TP biopsy cancer detection rate is comparable with other international centres (Table 7), showing cancer detection ranges of 35.0–76.0% and clinically significant cancer detection ranges of 33.0–60.1%. The cancer detection rate for 6% of our TP biopsy patients who had saturation biopsy was 50%. Hence, our increased prostate cancer detection rate in the TP cohort was unlikely confounded by patients who underwent saturation biopsies. Of note, none of the patients with clinically significant cancers with a PSA < 10 had a saturation biopsy performed. We postulate that this increase in detection rate could be, in part, due to a higher pick up rate in anterior zone cancers. However, we also recognise certain limitations on the comparison of cancer detection rates between our TP and TR cohorts. For example, the use of MRI prostate and number of cores sampled could not be controlled for.

**TABLE 7:** Freehand LA‐TP cancer detection rates

Freehand LA‐TP Bx	Sample size	Precision point device use	Biopsy‐naïve	Cores taken	PSA	Cancer detection rate	CS cancer detection rate (GG ≥ 2)
Hong Kong ‐ Lo et al.^14^	100	No	81%	‐	12.0 (7.5–25.7)	35.0%	‐
London, UK ‐ Kum et al.^12^	176	Yes	82%	‐	34 (15–157)	76.0%	57.6%
Taiwan ‐ Huang et al.^15^	130	No	100%	10 (10–10)	9.3 (6.3–20.3)	45.0%	‐
USA (Johns Hopkins) ‐ Meyer et al.^9^	43	Yes	72%	‐	6.1 (0.8–32.9)	48.0%	33.0%
USA (University Connecticut) ‐ Ristau et al.^8^	1000	Yes (117/1000)	74%	16 (14–20)	7.9 (5.5–11.9)	60.7%	40.3%
Toronto, Canada ‐ Stefanova et al.^7^	1287	No	‐	‐	7.05	49.8%	60.1%
Cambridge, UK ‐ Thurtle et al.^16^	30	No	0%	11 (10–12)	5.3 (0.72–36.9)	43.3%	‐
Singapore ‐ Chen et al.	212	Yes	94%	12 (4–38)	13.17 (6.82–47.13)	61.7%	51.9%

Abbreviations: CS, clinically significant; DRE, digital rectal examination; GG, group grade; LA‐TP, local anaesthesia‐transperineal; PSA, prostate specific antigen; UTI, urinary tract infection; VAS, visual analogue scale.

We also analysed the cancer detection rate of anterior zone tumours in our TP biopsy‐naïve cohort, where 49.5% had positive anterior cores of which a significant proportion (83.8%) having clinically significant cancer (≥GG2). It is recognised that anterior tumours are more difficult to biopsy using the TR approach, with false negative rates of up to 30%, attributed to inadequate sampling of these regions.^18^, ^19^, ^20^, ^21^ A previous study by Mabjeesh et al.^22^ evaluated patients with two or more previous negative transrectal ultrasonography (TRUS) biopsies who underwent TP biopsy. They had a 26% pickup rate, of which 83% had carcinomas detected in the anterior zone of the prostate. There is also an entity described by Lawrentschuk et al.^23^ as prostatic evasive anterior tumour syndrome (PEATS), which describes anterior zone prostatic carcinomas that have previously negative TRUS biopsies, manifesting as rising PSA. This, however, requires further validation as two separate meta‐analysis and systematic reviews recently comparing diagnostic outcomes and accuracies between the two methods showed no statistical difference in the detection of prostate cancer.^2^, ^24^

To assess the feasibility of this technique under LA, we also assessed patients' peri‐procedural pain score using the VAS. We started recording VAS scores after our initial learning curve. In total, we analyzed the VAS score of 112 of 212 patients. This was taken immediately after the patient had completed the TP biopsy and was asked to rate a pain score for the entire procedure. The mean VAS score was 3.67 ± 2.57 (0–9). Table 6 summarises the VAS scores (range = 2.75–4) in other centres performing LA TP biopsy. Whereas we asked patients for an overall pain score after completion of the biopsy, some studies stratified their pain data into different segments during the biopsy, including ultrasound probe insertion, LA administration and the biopsy itself. Kum et al.^12^ revealed pain scores at 1.05, 3.78 and 2.8 for probe insertion, LA administration and biopsy, respectively. Whereas we did not report pain scores in our TR biopsy cohort, there have been other studies comparing this. Guo et al. reported a statistically significant increased mean VAS score with TP compared with TR biopsy.^25^ This could be in part due to the penetration of the TP biopsy gun through the sensitive perineal skin and the surrounding neurovascular bundles encasing the prostatic capsule, unlike penetration of the rectal mucosa, which has been shown to have a lower sensitivity to pain.^26^ However, there have been more recent studies^27^ suggesting that the majority of patients who had previously undergone TR biopsy rated the TP approach more tolerable and some reporting no difference in perceived discomfort.

Given the described benefits, the TP approach has been gaining presence. However, it has yet to gather widespread adoption. Some barriers may be that most urologists were trained in TR biopsy and are not familiar with the transition to TP biopsy. Proper training is needed for urologists to learn TP biopsy under LA. In addition, there is additional capital cost in procuring the PrecisionPoint™ Transperineal Access System. However, the eventual shift towards TP biopsy seems to help drive overall cost down, with lower infectious‐related admissions.^28^ In the United Kingdom, the TRexit initiative,^29^ which comprises six London hospitals, have successfully ceased all TR biopsies and converted to a pure TP under LA biopsy service since March 2019. Our institution has also moved away from TR biopsies since October 2018 and is now exclusively performing TP biopsies.

In conclusion, TP biopsy under LA is safe and well tolerated. There is a lower complication rate, in particular sepsis, as compared with TR biopsy. There is an at least comparable cancer detection rate with TP compared with TR biopsies.

## CONFLICT OF INTEREST

None of the contributing authors have any conflict of interest, including specific financial interests or relationships and affiliations relevant to the subject matter or materials discussed in the manuscript.
